# The Association Between Different Types of Dietary Carbohydrates and Colorectal Cancer: A Case-Control Study

**DOI:** 10.3389/fnut.2022.898337

**Published:** 2022-07-12

**Authors:** Mona Jonoush, Soroor Fathi, Naeemeh Hassanpour Ardekanizadeh, Golsa Khalatbari Mohseni, Nazanin Majidi, Seyed Ali Keshavarz, Soheila Shekari, Shiva Nemat Gorgani, Saheb Abbas Torki, Mahtab Sotoudeh, Fatemeh Habibi, Maryam Gholamalizadeh, Atiyeh Alizadeh, Saeid Doaei

**Affiliations:** ^1^Department of Clinical Nutrition and Dietetics, Faculty of Nutrition and Food Technology, National Nutrition and Food Technology Research Institute, Shahid Beheshti University of Medical Sciences, Tehran, Iran; ^2^Department of Community Nutrition, School of Nutrition and Food Science, Isfahan University of Medical Sciences, Isfahan, Iran; ^3^Department of Clinical Nutrition, School of Nutrition and Food Sciences, Shiraz University of Medical Sciences, Shiraz, Iran; ^4^Nutrition and Metabolic Diseases Research Center, Ahvaz Jundishapur University of Medical Sciences, Ahvaz, Iran; ^5^Department of Nutrition, Science and Research Branch, Islamic Azad University, Tehran, Iran; ^6^Department of Clinical Nutrition, School of Nutritional Sciences and Dietetics, Tehran University of Medical Sciences, Tehran, Iran; ^7^Department of Nutrition, Faculty of Nutrition Sciences, Shiraz University of Medical Sciences, Shiraz, Iran; ^8^Department of Nutrition, Science and Research Branch, Islamic Azad University, Tehran, Iran; ^9^Cancer Research Center, Shahid Beheshti University of Medical Sciences, Tehran, Iran; ^10^Department of Pharmacognosy, Faculty of Pharmacy, Tehran University of Medical Sciences, Tehran, Iran; ^11^Department of Obstetrics and Gynecology, Reproductive Health Research Center, School of Medicine, Al-Zahra Hospital, Guilan University of Medical Sciences, Rasht, Iran

**Keywords:** colorectal cancer, dietary intake, carbohydrates, mortality, sociodemographic questionnaire

## Abstract

**Background:**

Several factors such as genetics and dietary intake are involved in the development of colorectal cancer (CRC). Higher intake of dietary carbohydrates may be associated with an increased risk of CRC. This study aimed to investigate the association between different types of dietary carbohydrates and CRC.

**Methods:**

This hospital-based case–control study was carried out from June 2020 to May 2021 on 480 randomly selected participants including 160 CRC patients and 320 healthy controls aged 35–70 years in Firoozgar hospital, Tehran, Iran. Dietary intake was assessed using Food Frequency Questionnaire (FFQ). Nutritionist IV software was used to determine the intake of calorie and various forms of dietary carbohydrates including total carbohydrate, simple sugar, glucose, fructose, galactose, sucrose, lactose, and maltose.

**Results:**

The average daily intake of calorie, carbohydrates, sugar, glucose, fructose, sucrose, and maltose were significantly higher among CRC cases compared to the controls (All *P* < 0.05). The logistic regression found significant associations between CRC with dietary intake of carbohydrates (OR = 1.009, CI 95%: 1.003–1.01, *P* = 0.002), sugar (OR = 1.02, CI 95%: 1.01–1.03, *P* < 0.001), glucose (OR = 1.06, CI 95%: 1.01–1.11, *P* = 0.009), fructose (OR = 1.31, CI 95%: 1.19–1.43, *P* < 0.001), sucrose (OR = 1.19, CI 95%: 1.12.−1.25, P < 0.001), maltose (OR = 9.03, CI 95%: 3.93–20.78, *P* < 0.001), galactose (OR = 1.31, CI 95%: 1.07–1.6, *P* = 0.008), and lactose (OR = 1.009, CI 95%: 1.01–1.18, *P* = 0.02). This association remained significant after adjustment for sex and age (except for galactose and lactose), and additional adjustment for sleep, tobacco, and alcohol level, and further adjustment for calorie intake and body mass index (BMI) (except for glucose).

**Conclusions:**

A positive association was found between CRC and dietary intake of carbohydrates, sugar, fructose, sucrose, and maltose. Following a low-carbohydrate, low-sugar diet may help prevent CRC. Future longitudinal studies are warranted to confirm these findings.

## Introduction

Colorectal cancer (CRC) is a malignant disease results from adenomatous polyps or adenomas in the colon and rectum (1). CRC is the third most common disease around the world and the second greatest cause of death, according to Global Cancer Incidence, Mortality, and Prevalence (GLOBOCAN) 2018 database (2, 3). CRC is the second and third most common cancer in Iranian women and men, accounting for 7 and 8 per 100,000 persons, respectively (4, 5) and the number of new CRC cases is predicted to be among cancers with the greatest increase from 2016 to 2025 (2).

Multiple factors, including demographic, genetics, lifestyle, and environmental factors are involved in the development of CRC. CRC occurs mostly in people aged 50 years or older, people with genetic susceptibility, and people with a family history of CRC (3–6). The non-hereditary or sporadic CRC is the most prevalent type of CRC (7, 8) and resulted from somatic mutations in response to environmental factors like obesity, daily drinking of alcohol, smoking, physical inactivity, and poor diet with high intake of red and processed meat and low intake of fruits, vegetables, and calcium (2, 9–11). One of the most important risk factors for CRC is reported to be a high intake of macronutrients which leads to excessive calorie intake and obesity (12).

Recent studies reported that a high intake of dietary carbohydrates may be associated with the increased risk of many health outcomes, such as all-cause mortality rate and many cancers such as CRC (13, 14). A high glycemic load (GL) diet such as the western diet which includes high amounts of refined carbohydrates (for example white rice and noodles) may promote CRC risk through its hyperinsulinemia effects (14–18).

The results of studies on the relationship between dietary carbohydrate intake and CRC risk is controversial. A recent study indicated that a higher intake of dietary carbohydrate may be a risk factor for CRC in men populations (19). Stewart et al. indicated that total intake of simple sugars (i.e., fructose, glucose, and sucrose) was associated with higher levels of inflammatory and angiogenesis biomarkers among CRC patients (20). Włodarczyk et al. reported that fructose is associated with tumorigenesis and the increased risk of CRC (21). However, some other studies reported that higher intake of dietary carbohydrate was a protective factor against CRC (15, 22, 23). Differences in the results on the relationship between dietary carbohydrates and CRC may be due to the different effects of different types of dietary carbohydrates on the development of CRC. Therefore, this study aimed to investigate the association between different types of dietary carbohydrates and CRC.

## Methods

### Study Population

This hospital-based case–control study was carried out from June 2020 to May 2021 on 480 randomly selected participants in the Firoozgar hospital, <city>Tehran</city>, Iran. The cases were eligible to be enrolled in this study if they were diagnosed with pathologically confirmed CRC (stages 3 and 4) within recent 3 months, aged 35–70 years, had no history of any diet-related diseases and did not take any drugs affected on dietary intake. The age-matched control group was selected from non-CRC patients referred to Firoozgar hospital for general check-ups. Inclusion criteria for the control group included consent to participate in the study, age between 35 and 70 years, no history of cancer or other diseases that affect dietary intake, and no use of drugs that affect dietary intake. The participants with misreported dietary intake and/or calorie intakes lower than 800 kcal/day or higher than 4,200 kcal/d were excluded. Finally, a total of 480 people including 160 CRC patients and 320 controls were included in this study. Trained interviewers collected data on demographic and socioeconomic status, dietary assessment, and physical activity *via* face to face interviews.

A general questionnaire was used to collect sociodemographic information of the participants including age, sex, education level, income level, marital status, medical history, and smoking status and alcohol consumption (more than 2–3 drinks per day) before CRC diagnosis. Participants' physical activity level was evaluated using a validated International Physical Activity Questionnaire (IPAQ) (25) and the results were expressed as metabolic equivalents (MET minutes a week).

### Anthropometric Measurements

Trained researchers did the measurements of Anthropometric indices. Weight was measured with a SECA weighing scale (Model 803, SECA Corp., Hamburg, Germany) to the nearest 0.1 kg, and height was measured using a SECA stadiometer with an accuracy of 0.5 cm. Body mass index (BMI) was measured as weight (in kilograms) divided by square of height (in meters).

### Dietary Assessment

Dietary intake was assessed through a validated 168-item semi-quantitative Food Frequency Questionnaire (FFQ) (24). The participants reported the amount of consumption of each food item in the last year before cancer diagnosis. The collected data was analyzed using the Nutritionist IV software and the intake of calorie, total carbohydrate, and different types of dietary carbohydrates including simple sugar, glucose, fructose, galactose, sucrose, lactose, and maltose were assessed.

### Statistical Analysis

To compare the status of socio-demographic factors, anthropometric measurements, and dietary intake among the case and control groups, the independent *T*-test and the chi-square test were used for quantitative and qualitative variables, respectively. The Shapiro-Wilk test was used to assess the distribution conformity of examined parameters with a normal distribution. Data on food intake was quantitatively analyzed and the association between dietary carbohydrates and CRC were estimated using binary logistic regression in four models including raw model (model 1), adjusted for age and sex (model 2), additionally adjusted for physical activity (as continues), smoking, and alcohol consumption (model 3), and further adjusted for BMI (as continues) and calorie intake (model 4). Statistical analyses were performed using SPSS version 20.0 (SPSS Inc., Chicago, USA). The results were considered statistically significant at *P*-value < 0.05. We did not adjust the *P*-value for multiple comparisons. *P*-value adjustments reduce the chance of making type I errors, but they increase the chance of making type II errors.

### Ethics Considerations

This study was approved by ethics review board of Shahid Beheshti University of Medical Sciences, Tehran, Iran (Code: IR.SBMU.CRC.REC.1398.028). After the description of the methodology and purpose of the study, a written consent form was collected from all participants.

## Results

The Shapiro-Wilk test showed that the data was normally distributed. The sociodemographic and lifestyle characteristics of CRC cases and controls are presented in Table 1. Smoking was significantly higher in the cases compared to the controls (7.6 vs. 0.8%, *P* = 0.003). The controls had significantly higher BMI compared to the cases (28.76 ± 3.97 vs. 27.58 ± 3.25 kg/m^2^, *P* < 0.001). There were no statistically significant differences between cases and controls with regards to age, gender, weight, alcohol intake, and physical activity.

**Table 1:** Characteristics of the participants.

	**Cases (*n* = 160)**	**Controls (*n* = 320)**	***P* ^#^**
Age (yr)	52.36 ± 17.06	51.8 ± 10.82	0.05
Males	81 (54/7%)	163 (51.1%)	0.11
Weight (kg)	69.39 ± 8.64	70.12 ± 10.59	0.44
Height (cm)	158.49 ± 7.59	156.11 ± 5.31	<0.001
BMI (kg/m^2^)	27.58 ± 3.25	28.76 ± 3.97	<0.001
Physical activity (h/wk)	7.51 ± 1.95	7.31 ± 1.58	0.28
Smokers^*^	2 (7.6%)	1 (0.8%)	0.003
Alcohol users^*^	0 (0.0%)	3 (1.2%)	0.73

*BMI, Body mass index. Quantitative and qualitative data are presented as mean ± SD and number (%), respectively*.

* *Data on smoking and alcohol consumption are related to the FFQ data collection period (before CRC diagnosis)*.

# *The analyses were performed using the independent T-test for quantitative variables and a chi-square test for qualitative variables*.

Table 2 presented a comparison of the dietary intake between the case and control groups. The average daily consumption for calorie (2,568.76 ± 404 vs. 2,493.38 ± 176 kcal/d, *P* = 0.006) and carbohydrates (368.88 ± 51.49 vs. 354.28 ± 33.72 g/d, *P* < 0.001) were significantly higher among CRC cases compared to the controls. Regarding to the different types of carbohydrates, the CRC cases had significantly higher intake of sugar (126.79 ± 31.57 vs. 116.06 ± 20.64 g/d, *P* = 0.001), glucose (19.01 ± 5.02 vs. 17.67 ± 4.59 g/d, *P* = 0.006), fructose (23.80 ± 6.01 vs. 21.30 ± 5.94 g/d, *P* = 0.001), sucrose (42.02 ± 13.31 vs. 36.98 ± 9.14 g/d, *P* = 0.001) and maltose (2.33 ± 1.1 vs. 1.82 ± 1.17 g/d, *P* = 0.001). There were no statistically significant differences between the cases and controls in the dietary intake of protein, galactose and lactose.

**Table 2:** Dietary intake of the participants^*^.

	**Cases (*n* = 160)**	**Controls (*n* = 320)**	***P* ^#^**
Calorie (kcal)	2,568.76 (±404.48)	2,493.38 (±176.03)	0.006
Protein (g/d)	85.77 ± 9.16	85.39 ± 19.85	0.78
Carbohydrates (g/d)	368.88 ± 51.49	354.28 ± 33.72	<0.001
Sugar (g/d)	126.79 ± 31.57	116.06 ± 20.64	0.001
Galactose (g/d)	4.13 ± 5.12	3.85 ± 2.74	0.44
Glucose (g/d)	19.01 ± 5.02	17.67 ± 4.59	0.006
Fructose (g/d)	23.80 ± 6.01	21.30 ± 5.94	0.001
Sucrose (g/d)	42.02 ± 13.31	36.98 ± 9.14	0.001
Lactose (g/d)	14.24 ± 12.93	12.92 ± 4.98	0.12
Maltose (g/d)	2.33 ± 1.1	1.82 ± 1.17	0.001

* *Data are presented as mean ± SD*.

# *The analyses were performed using the independent T-test for quantitative variables and a chi-square test for qualitative variables*.

Table 3 presents the logistic regression models of the association between CRC and dietary carbohydrates. Dietary intake of carbohydrates (OR = 1.009 CI 95%: 1.003–1.01, *P* = 0.002), sugar (OR = 1.02 CI 95%: 1.01–1.03, *P* < 0.001), glucose (OR = 1.06 CI 95%: 1.01–1.11, *P* = 0.009), fructose (OR = 1.31 CI 95%: 1.19–1.43, *P* < 0.001), sucrose (OR = 1.19 CI 95%: 1.12–1.25, *P* < 0.001), maltose (OR = 1.14 CI 95%: 1.03–1.23, *P* < 0.001), galactose (OR = 1.31 CI 95%: 1.07–1.6, *P* = 0.008) and lactose (OR = 1.009 CI 95%: 1.01–1.18, *P* = 0.02) were associated with CRC. This relationship remained significant after adjusting for sex and age (except for galactose and lactose) (Model 2), after additional adjustment for physical activity, sleep, tobacco, and alcohol level (Model 3), and after further adjustment for calorie intake and BMI (except for glucose)) Model 4).

**Table 3:** Logistic regression of the association between colorectal cancer and carbohydrate intake.

	**Model 1**		**Model 2**		**Model 3**		**Model 4**	
	**OR (CI 95%)**	***P***	**OR (CI 95%)**	***P***	**OR (CI 95%)**	***P***	**OR (CI 95%)**	***P***
Carbohydrates	1.009 (1.003–1.01)	0.002	1.01 (1.005–1.01)	0.001	1.02 (1.01–1.03)	<0.001	1.02 (1.02–1.03)	0.011
Sugar	1.02 (1.01–1.03)	<0.001	1.02 (1.01–1.03)	<0.001	1.07 (1.04–1.1)	<0.001	1.06(1.03–1.09)	<0.001
Galactose	1.31 (1.07–1.6)	0.008	1.27 (0.97–1.66)	0.08	1.25 (0.94–1.67)	0.11	1.38 (0.96–1.97)	0.07
Glucose	1.06 (1.01–1.11)	0.009	1.08(1.02–1.14)	0.005	1.25 (1.08–1.45)	0.003	1.15 (0.98–1.35)	0.06
Fructose	1.31 (1.19–1.43)	<0.001	1.36 (1.19–1.55)	<0.001	1.35 (1.17–1.55)	<0.001	1.29 (1.11–1.49)	0.001
Sucrose	1.19 (1.12.-1.25)	<0.001	1.2 (1.12–1.29)	<0.001	1.23 (1.13–1.33)	<0.001	1.2 (1.11–1.31)	<0.001
Lactose	1.09 (1.01–1.18)	0.02	1.06 (0.95–1.17)	0.24	1.06 (0.95–1.19)	0.24	1.04 (0.92–1.17)	0.49
Maltose	1.14 (1.03–1.23)	<0.001	1.35 (1.08–1.62)	<0.001	1.62 (1.05–1.71)	<0.001	1.82 (1.12–1.82)	0.001

*Model 1: Crude, Model 2: Additionally adjusted for sex and age. Model 3: Additionally adjusted for physical activity, sleep, tobacco, and alcohol Level. Model 4: Additionally adjusted for calorie intake and body mass index (BMI)*.

## Discussion

The results of the present study indicated that the case group had a significantly higher intake of calories, carbohydrates, sugar, glucose, fructose, sucrose, and maltose. This study found a positive association between CRC and dietary intake of carbohydrates, sugar, fructose, sucrose, and maltose after adjusting the confounder factors. In line with this study, a meta-analysis of 17 observational studies (846004 healthy participants and 14402 CRC patients) on the association between dietary carbohydrate intake and CRC risk found that higher dietary carbohydrate intake may be a risk factor for CRC risk in men (19). The link between cancer and dietary carbohydrate intake may be different in women than in men. However, in the present study, we were not able to investigate the association between CRC and carbohydrate intake in men and women separately due to sample size limitations.

On the other hand, Howarth et al. reported that carbohydrate intake appear to protect against CRC in women with a rice-based diet (15). However, differences between different types of dietary carbohydrates were not assessed in this study. Also, Cho et al. (22) reported that dietary intakes of glucose, fructose, galactose, sucrose, maltose, lactose, and total sugars were not significantly associated with the prevalence of colorectal adenoma. This finding could be due to the fact that data was collected using self-administered questionnaires and the patients may intentionally underestimate their carbohydrate intake. Huang et al. (25) in a case-control study examined the relationship between total carbohydrates, non-fiber carbohydrates, total fiber, starch, dietary glycemic index (GI) and glycemic load (GL) with the risk of CRC on 1,944 CRC cases and 2,027 controls. The results indicated that there was no association between total carbohydrate intake and the risk of CRC. Dietary fiber was associated with a 53% reduction in the risk of CRC and dietary GI was also positively associated with the risk of CRC. However, in this study, the association of cancer with different types of mono and disaccharides was not investigated. Also, in our study, the status of dietary fiber intake and glycemic index were not examined, which may affect the accuracy of the results.

The exact mechanisms of the effect of dietary carbohydrates in CRC pathogenesis are not clear. The main pathway of glucose metabolism in cancer cells is aerobic glycolysis (which is called Warburg effect) and both glycolysis and mitochondrial metabolisms are crucial to cancer cells (22). In cancer cells, glucose uptake and the production of lactate are dramatically increased, even in the presence of oxygen and fully functioning mitochondria. Cancer cells are glucose-dependent and this classic type of metabolic change provides substrates required for cancer cell proliferation and division, which eventually can lead to tumor growth and metastatic progression (26). Moreover, fructose is linked with tumorigenesis and increased risk of CRC. Fructose is absorbed from the gastrointestinal tract by members of the glucose transporter (GLUT) family. Fructose can be utilized by tumor cells as an alternative energy source to maintain proliferation and exert chemotherapy resistance *in vitro* by upregulating GLUT5 (8). The GLUT5 gene expression was found in 96.7% of cancer specimens and only in 53.3% of healthy samples (21). Moreover, Stewart et al. indicated that intake of simple sugars including fructose, glucose, and sucrose was associated with higher levels of inflammatory and angiogenesis biomarkers among CRC patients. They hypothesized that inflammation and angiogenesis may be involved in the link between sugar consumption and CRC and may lead to increased recurrence, promoted proliferation and survival of malignant cells, and decreased survival rates (20). Further studies are needed to identify the mechanism of the effects of different types of carbohydrates on tumorigenesis.

The strengths of this study were its adequate sample size compared with other case-control studies on diet in CRC patients, performed on Iranian people with colon cancer (who have been less studied with this regard), focus on participants' dietary intake prior to diagnosis to identify possible predictive role of dietary intake of carbohydrates in CRC, and highlighting the differences between the association of different types of carbohydrates with CRC. However, this study had some limitations. The first limitation is the using of self-report FFQ which has potentially a recall bias. Second, there are several other factors that may affect the results. However, the effects of some confounders such as age, sex, smoking, tobacco, and BMI were adjusted, but the effects of some other factors, such as genetics, psychological status, and dietary intake of other food groups such as dietary fibers and meats were not assessed. Third, due to the limited sample size, different types of CRC, including colon cancer and rectal cancer, were not evaluated separately.

## Conclusion

In conclusion, the results of this case-control study indicated that the patients with CRC had a significantly higher intake of calorie, carbohydrates, sugar, glucose, fructose, sucrose and maltose. A positive association was found between CRC and dietary intake of carbohydrates, sugar, fructose, sucrose, and maltose. Following a low-carbohydrate, low-sugar diet may help prevent CRC. Future longitudinal studies are needed to verify these findings and discover the underlying mechanisms of the possible association of different types of carbohydrates and CRC.

## Data Availability Statement

The raw data supporting the conclusions of this article will be made available by the authors, without undue reservation.

## Ethics Statement

The studies involving human participants were reviewed and approved by IR.SBMU.PHNS.REC.1399.038. The patients/participants provided their written informed consent to participate in this study.

## Author Contributions

MJ, SD, SAK, SF, GKM, NM, SNG, and MG designed the study and carried out the data collection. SAT, MS, FH, SS, NHA, AA, and SD were involved in the analysis of the data, and critically reviewed the manuscript. This study was conducted at the Shahid Beheshti University of Medical Sciences, Tehran, Iran. All authors contributed to the article and approved the submitted version.

## Funding

Funding for this study was provided by Cancer Research Center of Shahid Beheshti University of Medical Sciences, Tehran, Iran (Code 43002312).

## Conflict of Interest

The authors declare that the research was conducted in the absence of any commercial or financial relationships that could be construed as a potential conflict of interest.

## Publisher's Note

All claims expressed in this article are solely those of the authors and do not necessarily represent those of their affiliated organizations, or those of the publisher, the editors and the reviewers. Any product that may be evaluated in this article, or claim that may be made by its manufacturer, is not guaranteed or endorsed by the publisher.
